# Liver injury in COVID-19: an insight into pathobiology and roles of risk factors

**DOI:** 10.1186/s12985-024-02332-y

**Published:** 2024-03-15

**Authors:** Abbas Tazarghi, Sahar Bazoq, Mohammad Hosein Taziki Balajelini, Mohsen Ebrahimi, Seyed Mehran Hosseini, Hadi Razavi Nikoo

**Affiliations:** 1 Department of Microbiology, Faculty of Medicine, Golestan University of Medical Sciences, Gorgan, Iran; 2 Department of Otorhinolaryngology, Neuroscience Research Center, School of Medicine, Golestan University of Medical Sciences, Gorgan, Iran; 3 Neonatal and Children’s Health Research Center, Golestan University of Medical Sciences, Gorgan, Iran; 4 Department of Physiology, School of Medicine, Neuroscience Research Center, Golestan University of Medical Sciences, Gorgan, Iran; 5 Infectious Diseases Research Center, Golestan University of Medical Sciences, Gorgan, Iran

**Keywords:** *SARS-CoV-2*, COVID-19, Liver injury, Risk factors, Pathobiology mechanisms

## Abstract

COVID-19 is a complex disease that can lead to fatal respiratory failure with extrapulmonary complications, either as a direct result of viral invasion in multiple organs or secondary to oxygen supply shortage. Liver is susceptible to many viral pathogens, and due to its versatile functions in the body, it is of great interest to determine how hepatocytes may interact with *SARS-CoV-2* in COVID-19 patients. Liver injury is a major cause of death, and *SARS-CoV-2* is suspected to contribute significantly to hepatopathy. Owing to the lack of knowledge in this field, further research is required to address these ambiguities. Therefore, we aimed to provide a comprehensive insight into host-virus interactions, underlying mechanisms, and associated risk factors by collecting results from epidemiological analyses and relevant laboratory experiments. Backed by an avalanche of recent studies, our findings support that liver injury is a sequela of severe COVID-19, and certain pre-existing liver conditions can also intensify the morbidity of *SARS-CoV-2* infection in synergy. Notably, age, sex, lifestyle, dietary habits, coinfection, and particular drug regimens play a decisive role in the final outcome and prognosis as well. Taken together, our goal was to unravel these complexities concerning the development of novel diagnostic, prophylactic, and therapeutic approaches with a focus on prioritizing high-risk groups.

## Background

Severe acute respiratory syndrome coronavirus-2 (*SARS-CoV-2*) is the most recently discovered member of the family *Coronaviridae* and is responsible for causing the coronavirus disease (COVID-19). The *SARS-CoV-2* outbreak first emerged in Wuhan, China, in December 2019 and rapidly erupted into a pandemic with millions of laboratory-confirmed cases and deaths worldwide [1]. *SARS-CoV-2* is a helical, pleomorphic, enveloped virus (diameter: 70–90 nm) containing a non-segmented, positive-sense, single-stranded RNA genome of ~ 30 kb with a 5’-cap at the beginning and a 3’-poly-A tail at the end. The genome contains 14 open reading frames (ORFs) responsible for coding at least 26 structural, non-structural, and accessory proteins. The clinical manifestations of COVID-19 range from an asymptomatic or mild disease (81%) to severe complications (14%), fatal pneumonia, multiorgan failure (MOF), and death (5%) [2, 3]. *SARS-CoV-2* mainly targets bronchial epithelial cells and pneumocytes (cellular tropism) to initiate its replication in the lower to deepest areas of the respiratory system, such as the trachea and lungs (tissue tropism). The reason for this tropism is considered to be the presence of many viral and cellular factors that can interact with and control the early stages of viral reproduction [4]. However, some studies have reported that 60–70% of patients complain of extrapulmonary sequelae [3], including thrombosis, embolism, myocardial dysfunction and arrhythmia, kidney injury, gastrointestinal symptoms, hepatopathy, ocular complications, neurologic illnesses, and dermatologic problems [5].

Liver is one of the frequently affected organs in COVID-19 patients, and the level of damage inflicted on it is associated with the severity of the illness. Hereafter, we refer to this condition as *SARS-CoV-2*-induced hepatopathy (SIH). SIH can be a leading cause of death, particularly in patients with underlying liver diseases [6]. According to clinical data, SIH engages more than 50% of COVID-19 patients, from which 15–65% of cases experience abnormalities in liver biochemistries [7]. The detailed mechanism of SIH is not yet fully understood; therefore, in this review, we focused on the evaluation of available information concerning the different molecular and cellular mechanisms involved in SIH. We hope that this review illuminates the path for further investigations regarding the associated liver pathogenesis, design of advanced drugs, more reliable detection methods, and updating of prognostic markers.

## An overview of how *SARS-CoV-2* can infect hepatocytes

Liver is a vital organ due to its pivotal role in the body, and the presence of certain receptors on its cells contributes to the attachment and penetration of *SARS-CoV-2* into hepatocytes [8]. *SARS-CoV-2* appears to target cells that highly express the membrane protein angiotensin-converting enzyme 2 (ACE-2) through the binding of its viral spikes (S-proteins) to these receptors. The S-protein has been observed to have two domains, S1 (containing the receptor binding domain aka RBD) and S2, which contribute to the attachment of the virus to its receptor and induce fusion, respectively [3, 9, 10]. ACE-2 is an enzyme that can be found either in soluble form or as a receptor bound to the surface of particular cells with a key role in the regulation of blood pressure and electrolyte homeostasis via renin-angiotensin-aldosterone system abbreviated as RAAS aka RAS [11]. Single-cell RNA sequencing (scRNA-seq) revealed that cholangiocytes have a high level of ACE-2 expression which can contribute to bile duct disorders and SIH [12]. *SARS-CoV-2* has also been shown to propagate successfully in hepatocellular carcinoma-derived cell lines Huh-7 and HepG2. These cells have been experimentally proven to have the highest expression level of ACE-2 among other cells, such as H460, MRC5, U251, and HEK293T [13]. Accordingly, the viral genome was also detected in the hepatocytes and cholangiocytes of COVID-19 patients, which supports the in vitro observations [14, 15]. It is noteworthy to bear in mind that underlying liver disorders, steatohepatitis, viral hepatitis, and hepatocellular carcinoma (HCC) might also be compelling enough to prompt SIH by elevating the hepatic level of ACE-2 expression [16].

In fact, it appears that there exists a mutual relationship between COVID-19 and liver injury, meaning that *SARS-CoV-2* can be a causative agent for the development of hepatopathy, while a pre-existing advanced liver disorder can also increase the morbidity and mortality rate of COVID-19. Studies have proposed that the incidence of liver injury in individuals with severe SARS and COVID-19 is notably higher (74.4%, 36.2%) in comparison with cases that had only a mild disease (43%, 9.6%), respectively. In addition, 58.06 to 78% of COVID-19 patients who died during hospitalization had different signs of liver complications, and the number of those who began to show abnormal liver function tests increased significantly (76.3% of 417 patients) after the second week of illness [17, 18].

## Why ACE-2 is the cornerstone

**Fig. 1:**
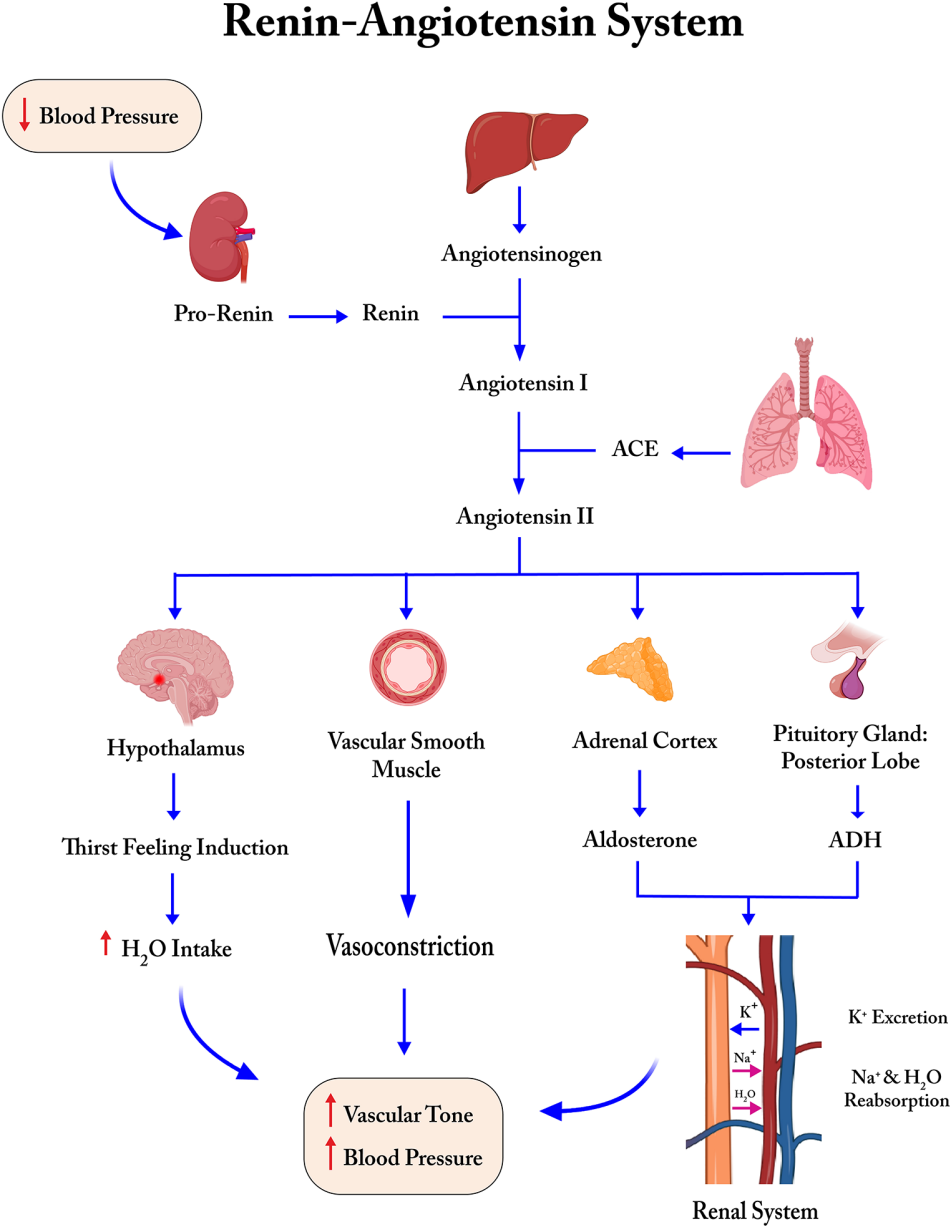
An overview of the renin-angiotensin-aldosterone system, including organs, enzymes, functions, interactions, and the ultimate outcome that leads to the modulation of blood pressure in response to situations in which the human body needs to alter the balance of electrolytes and its vascular tone

The human body applies a complex multimechanism-based process to compensate for hypotension and maintain its normal vascular tone when experiencing excessive loss of blood, stress, fight-flight response, diarrhea and dehydration, and strenuous physical activity [19]. RAAS is comprised of kidneys, liver, systemic vasculature, lungs, adrenal glands, pituitary gland, and hypothalamus, all of which cooperate to temporarily elevate the blood pressure when needed. Kidneys synthesize the enzyme/hormone renin from its precursor, prorenin, and secrete it into the blood flow. Renin, also known as angiotensinogenase, cleaves its substrate, angiotensinogen, which is secreted from the liver and converts it to angiotensin 1 (ANG-1). ANG-1 is further catalyzed by a receptor with enzymatic activity in the lungs called angiotensin-converting enzyme (ACE, aka ACE-1) and turns into angiotensin 2 (ANG-2). ANG-2 is a potent vasoconstrictor that adjusts electrolyte homeostasis and raises vascular tone; however, if its production and effects continue uncontrollably, it causes undesired hypertension, systemic inflammation, vessel linings injury, oxidative stress, fibrosis, apoptosis, and coagulation problems [20]. Here, ACE-2 acts as a negative regulator of ANG-2 and cleaves it into ANG 1–7, a vasodilator, to terminate the unfavorable consequences (Fig. 1). It may also compete with ACE to convert ANG-1 to ANG 1–9, the biological properties of which is an area of research. In COVID-19, a large group of organs, including the liver, are prone to serious damage when ACE-2 receptors are already occupied by *SARS-CoV-2* and cannot function properly [11].

## Liver biochemistry abnormalities in COVID-19

In general, liver injury is detected when alanine aminotransferase (ALT) and aspartate aminotransferase (AST) levels exceed three times, and alkaline phosphatase (ALP), total bilirubin (TBIL), and γ-glutamyltransferase (GGT) levels exceed two times the upper limit of normal values [21, 22]. This condition is termed transaminasemia, and in case of persistence for less than six months, it is referred to as “acute”, whereas any longer continuation is known as “chronic”, which needs critical attention [23]. There are several types of damage including hepatocellular, autoimmune, cholestatic, and infiltrative complications, each of which has its unique marker changes. The hepatocellular form is determined by the isolated or predominant elevation of serum transaminases, whereas in the cholestatic and infiltrative forms, the serum ALP or GGT levels are primarily elevated with a normal or light increase in serum transaminases. An elevated level of bilirubin in the cholestatic pattern can be used further to discern it from the infiltrative form [24]. Hypoalbuminemia (< 34 g/L), a non-specific marker of liver disease, has also been demonstrated to be associated with a deteriorated condition of COVID-19 [25]. Moreover, injury to bile duct cells has been observed in patients with COVID-19, accompanied by abnormally high GGT and ALP levels [12]. According to recent clinical studies, the escalation of ALT, AST, and GGT in these patients was 9.6–37.6%, 14.8–36%, and 13–24.4%, respectively, and there are supporting findings that *SARS-CoV-2* infection along with higher levels of plasma AST is associated with a greater risk of serious COVID-19 or even death. Other studies have also indicated that patients with elevated ALT, AST, and TBIL levels had a higher rate of admission to the intensive care unit (ICU), while mild cases showed a lower level of these markers with less severe consequences. Overall, it should be taken into consideration that the impact of SIH on COVID-19 patients might be dissimilar based on the stage of the disease and other factors mentioned earlier [26–28].

## Direct or indirect? That’s the question

The presence of a proper receptor does not guarantee that the infection certainly leads to productive replication since many other intracellular factors are also required to support the virus life cycle [29]. Liu et al. demonstrated that the profusion of ACE-2 varied among different organs, and there might be a direct relationship between the severity of organ dysfunction and the level of ACE-2 profusion in COVID-19. This study investigated the expression of viral proteins within different cells in the human body and reported that a large group of cells carried ACE-2, but only some of them were capable of expressing S-proteins. ACE-2-containing cells in the brain were found to lack the capability to support such co-localization in cases of *SARS-CoV-2* infection. The cells of the large intestine and renal proximal tubules, which express ACE-2, also contained only a few viral antigens, and surprisingly, none was observed in liver cells [9].

These observations imply that the pathogenesis of SIH might be secondary to other dysfunctions that compromise some organs, such as the heart and lungs, which control the distribution of blood and oxygen to the liver. On the other hand, there are findings proposing that hepatocytes (hepatocellular type) and biliary epithelial cells (cholestatic type) may also be directly prone to viral attack due to either the virus-specific protein 7a which induces apoptosis and cytopathic effect (CPE), or as a result of an immune response similar to that in human hepatitis B (HBV)-associated liver pathogenesis [21, 30, 31].

**Fig. 2:**
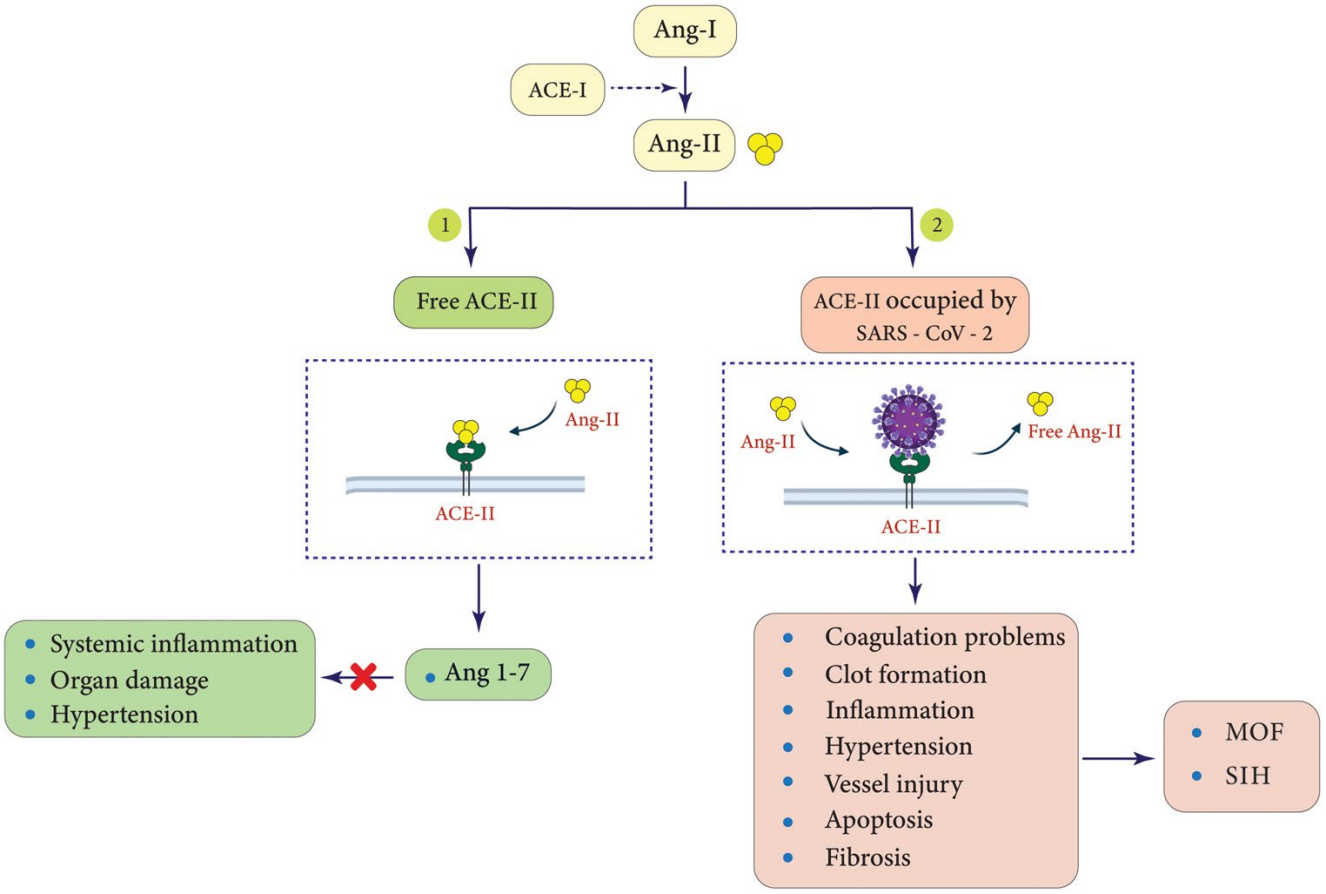
A scheme of ACE-2 receptors illustrating a comparison between two states: (*1*) the free to perform normally and (*2*) the exploited status by *SARS-CoV-2* which results in a variety of complications in patients with COVID-19

In addition, RAAS has been well recognized as a physiological scaffold responsible for the modulation of the cardiovascular, renal, and pulmonary systems, with ACE-2 functioning as a key negative regulator. Therefore, ACE-2 dysfunction can play a crucial role in the pathogenesis of many diseases (Fig. 2) [11, 32]. Of note, S-proteins decrease ACE-2 at both protein and mRNA levels through internalization, transcriptional, post-transcriptional, and intracellular pathways, which account for other mechanisms that contribute to multiorgan injury, including the liver [33]. Moreover, other studies have suggested that S-protein-binding proteins such as neuropilin 1 (NRP-1) and basigin (BSG) increase cellular infectivity probably by acting as a co- and alternative receptor for *SARS-CoV-2*, respectively. Additionally, transmembrane protease serine 2 (TMPRSS-2), endosomal cysteine proteases cathepsin L (CTSL) and B (CTSB), as well as paired basic amino acid cleaving enzyme (PACE aka FURIN) can act as putative priming receptors and play an essential role in catalyzing the S-protein for an effective attachment to ACE-2 at the early stages of cellular infection [29, 34, 35]. On the other hand, some studies concluded that not all the mentioned proteins, such as BSG, could be considered a replacement for ACE-2 because it lacked the required features to interact with the recombinant S-proteins [36]. In other words, the absence, inadequacy, or abundance of a certain receptor on a particular cell type does not necessarily exclude it from or include it within the tropism zone of *SARS-CoV-2*, and cellular infection is the net outcome of multiple factors that cooperate unitedly to facilitate the virus entry.

## Cytokine release syndrome

Following *SARS-CoV-2* infection, an acute respiratory distress syndrome (ARDS) might occur and trigger a massive release of pro-inflammatory and pro-thrombotic cytokines. This is referred to as cytokine release syndrome (CRS) or cytokine storm, and is a major cause of lung impairment and MOF. Therefore, as a result of an acute and excessive immune response, *SARS-CoV-2* induces inflammation, damaging not only the liver but also lungs, heart, kidneys, etc. [37]. It has also been demonstrated that individuals with higher levels of inflammatory markers, such as C-reactive protein (CRP), serum ferritin, lactate dehydrogenase (LDH), D-dimer, tumor necrosis factor-alpha (TNF-α), interleukin 6 (IL-6), and IL-2, are more likely to develop an insidious type of the disease accompanied by SIH. In brief, CRS in COVID-19 highlights the risk of MOF, and the chances of these patients receiving liver damage are noticeable unless timely therapeutic measures are taken to help the inflammation subside [37, 38].

## Ischemic and hypoxic liver injury in COVID-19

**Fig. 3:**
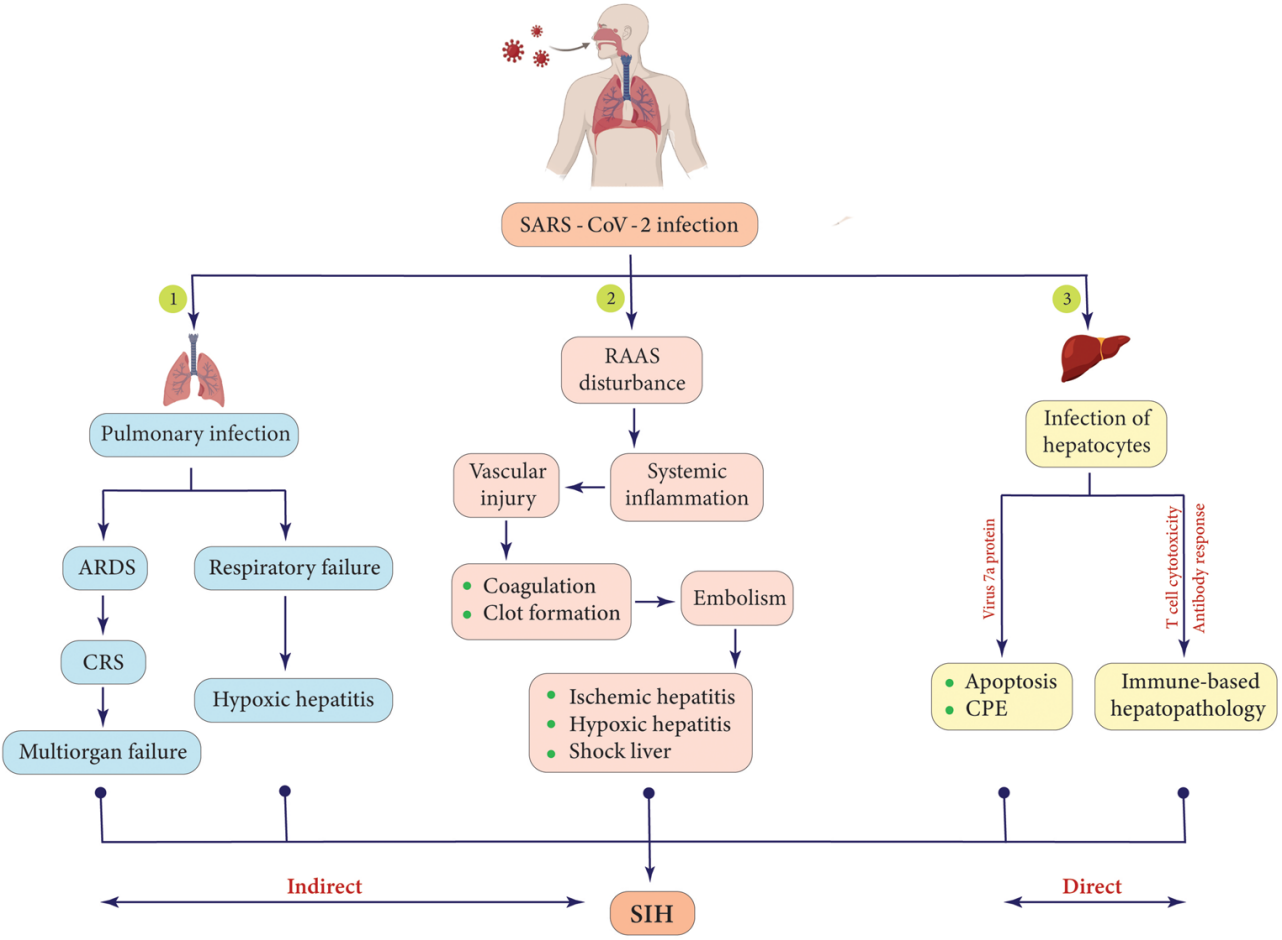
A depiction of various mechanisms that may contribute to the development of SIH in COVID-19 patients. It is noteworthy that, in addition to the direct invasion of *SARS-CoV-2* to ACE-2-positive hepatocytes, indirect pathways such as immune-based hepatopathology, cytokine storm, and RAAS disturbance should also be highlighted as potential mechanisms of SIH pathogenesis

COVID-19 is the possible cause of two other complications termed hypoxic hepatitis and ischemic hepatitis. Hypoxia is a condition in which the whole body (generalized) or part of it (local) suffers from deprivation of adequate oxygen supply to the tissues. On the other hand, ischemia is defined as the restriction or reduction of blood flow in a region of the body, which leads to hypoxia, too, due to oxygen shortage [39]. Epidemiological findings from many clinical studies provide evidence that S-proteins trigger the clotting process due to the induction of inflammation, both locally and systemically, which can contribute to the reduction or blockage of blood flow to the liver, resulting in lipid metabolism disorders, depletion of glycogen, lack of oxygen supply that leads to the hypoxic form as well, depletion of adenosine triphosphate (ATP), and ultimately the disturbance of hepatocytes. These alterations can result in shock liver, a life-threatening medical condition in which hepatocytes continue to lyse until the liver fails to operate properly. Shock liver is possibly provoked by the net effect of MOF and is determined by a sharp increase in aminotransferases to more than 20 times the upper limits [40–43]. Hypoxic/ischemic hepatitis may be closely related to the mortality rate of COVID-19 since patients who were admitted to the ICU had significantly abnormal hepatic function tests [44]. Taken together, hypoxic and ischemic hepatitis seem to be two of the major outcomes or causative mechanisms involved in SIH; however, further investigation is needed to discover the unknowns (Fig. 3).

## Oxidative stress and NETosis

COVID-19 has also been implicated in the development of oxidative stress. Oxidative stress is a condition in which the body fails to neutralize reactive oxygen species (ROS) and their harmful effects on different organs, including the liver. ROS are highly reactive molecules produced as byproducts during metabolism, and upon excessive accumulation, they can activate platelets and stimulate the coagulation cascade, which underpins the pathobiology of ischemic/hypoxic liver injury. Additionally, ROS can trigger certain intracellular pathways such as nuclear factor-kappa B (NF-κB), leading to the production of pro-inflammatory cytokines (pro-IL-1β, pro-IL-6, pro-IL-18, and TNF-α) involved in CRS and pyroptosis. That aside, NETosis is another molecular mechanism that protects the host as a part of the innate immune system. It is a unique type of cell death specific to neutrophils, and when prompted, a matrix of chromatins, histones, granular, and antimicrobial proteins, termed neutrophil extracellular traps (NETs), are produced and released to ensnare various pathogens, such as bacteria, viruses, and fungi. NETosis is provoked by several stimuli, including *SARS-CoV-2* sepsis, and despite its beneficial effects, an exaggerated continuation of it has been proposed to be associated with cytokine storms, endothelial dysfunction, formation of blood clots, MOF, and probably SIH [45, 46].

## Risk factors of liver injury in COVID-19 patients

**Fig. 4:**
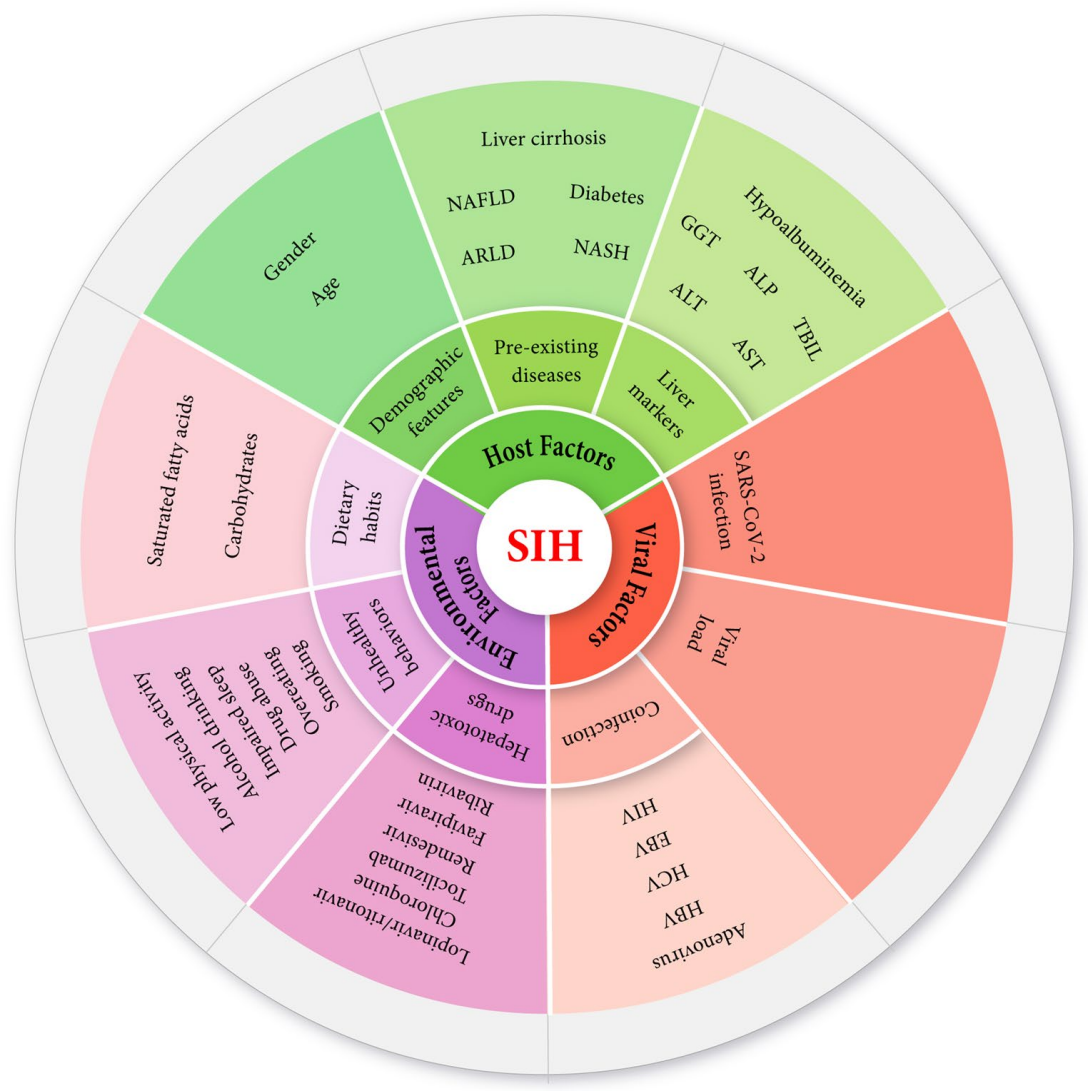
As a multifactorial complication, the development and severity of SIH are considered to be relatively dependent on host-oriented, environmental, and viral risk factors, which can influence the outcome of the disease in COVID-19 patients

SIH is a multifactorial complication, and there appear to be many risk factors linked to it. Although the available findings may not suffice to cover certain aspects of SIH in detail, we have classified the possible risk factors into three categories: host factors, environmental factors, and viral factors. Host factors point to a group of individual-oriented variables, such as age, gender, pre-existing diseases, and liver markers, while environmental and viral factors embrace dietary habits, unhealthy behavior, drugs, mutations, coinfection, etc. (Fig. 4).

### Host factors

The general health of a host is a conclusive criterion that determines an individual’s capacity to control and eliminate any infection in the body. Vigorous general health status can reinforce the immune system’s protection against COVID-19 and SIH. We assume that aging, male sex, and pre-existing medical conditions such as hypertension, liver and heart diseases, diabetes, chronic kidney disorders, obesity, chronic obstructive pulmonary disease (COPD), brain and nervous system conditions, dementia, a history of stroke, cancer, and blood disorders are among the most important culprits that can exacerbate the morbidity of COVID-19 (Table 1) [47].

**Table 1:** Host-oriented risk factors demonstrated in classes, types, and prognostic values, as well as the possible mechanisms and outcomes that may contribute to SIH in patients with COVID-19

Risk factors			Prognostic value	Possible mechanisms & outcomes	Reference
Category	Class	Type			
Host factors	Demographic features	Age	> 65 years	- Immunopathogenesis - Comorbidity - Decrease of stem cells - Higher expression of ACE-2 & TMPRSS-2	1, 47–51
		Gender	Male sex	- Sex hormones - Higher expression of ACE-2 - Sex-specific features of immune system	47, 52, 53
	Pre-existing diseases	ALD		- Alcohol-related steatohepatitis - Liver fibrosis & cirrhosis - Increase of inflammatory cytokines	54
		NAFLD / NASH		- Chronic inflammation - Hypercoagulability - Oxidative stress - Liver fibrosis - Liver cirrhosis - Hepatonecrosis - Longer clearance time of viruses	40, 56, 63
		Liver cirrhosis		- Ischemic/hypoxic liver injury - Hepatic encephalopathy - HCC	7, 16, 40, 54, 57–59
		Liver transplantation		- Higher chances of critical COVID-19 as a result of immunosuppression therapy	55
		Diabetes		- Higher expression of ACE-2 & FURIN - Lymphocytopenia - Elevation of IL-6	60
	Liver markers	ALT & AST	>3-fold of the upper limit		21, 22
		GGT	>2-fold of the upper limit		
		ALP & TBIL	>2-fold of the upper limit		
		Hypoalbuminemia	<34 g/L		25

It is difficult to accurately determine the age distribution of COVID-19 patients. However, it is assumed that aging influences the pathophysiology of the disease in several ways, and adults older than 65 years are more prone to SIH [1, 48]. There are also reports that neonates infected with *SARS-CoV-2* have lower chances of experiencing liver injury, implying that SIH might correlate with immunopathological factors and that immature adaptive immunity may be more advantageous in this context [49]. From the perspective of science, there are two types of aging systems termed chronological age and biological age. Chronological age, aka phenotypic age, is calculated merely based on the number of years that have passed since a person’s birthday, whereas biological age, aka epigenetic age, is defined on the basis of DNA methylation patterns and takes into account a collection of more factors, including nutrition, lifestyle, genetics, and diseases to display a more accurate picture of our overall body age. It is believed that the coexistence of underlying diseases is more likely to be seen in elderly people either chronologically or biologically. Furthermore, pulmonary stem cells undergo aging-induced degradation over time, which highlights the possibility of respiratory failure and developing SIH via hypoxic hepatitis [1, 50]. In addition, levels of ACE-2 and TMPRSS-2 expression are apparently higher in elderly individuals, explaining why aging is correlated with a higher risk of serious illness. Interestingly, analysis of epigenetic clocks and telomere shortening in two groups of people, including patients with severe and non-severe COVID-19, revealed that an increasing age acceleration occurs during the initial phases of the disease. Although this phenomenon can be partially reversed in later phases, *SARS-CoV-2* infection might expedite biological aging, which on a larger scale, unfavorably affects whole-body organs, including the liver [51].

It has also become clear that the risk of developing SIH is probably sex-biased [48]. According to epidemiological studies, males constitute up to 75% of all COVID-19 deaths, with three times the odds of being admitted to the ICU and a mortality rate at least 15% higher than that of females. Males showed a higher risk of organ dysfunction that may involve the liver when infected with *SARS-CoV-2*. It appears that higher expression levels of ACE-2 in men, sex-specific immunological differences driven by sex hormones, and the X chromosome can be the potential culprits to blame. Moreover, lifestyle and behavioral differences between females and males might be a case as well, because the latter exhibit greater tendencies for smoking, drinking, and irresponsibility toward wearing face masks, frequent handwashing, staying at home, etc. [52, 53].

Moreover, patients with chronic liver diseases (CLDs) are more likely to exhibit a critical form of COVID-19. Liver dysfunction, if not caused by *SARS-CoV-2* itself, might be a result of different conditions, including alcoholic liver disease (ALD), non-alcoholic fatty liver disease (NAFLD), non-alcoholic steatohepatitis (NASH), chronic or end-stage cirrhosis, and liver transplant complications. These states are among the significant preceding factors that can remarkably increase the severity of COVID-19 accompanied by hepatonecrosis [54–56]. COVID-19 patients with liver dysfunction, HCC, or cirrhosis experience a greater degree of injury to the liver. Such a complex medical history, along with higher hepatic expression of ACE-2 and TMPRSS-2 in critically ill patients, may generate immune system defects, coagulation disorders, renal failure, systemic inflammation, and hepatic encephalopathy [16, 40, 57, 58]. Reportedly, the lethal combination of cirrhosis and *SARS-CoV-2* infection has a poor prognosis, and the mortality rate in this group of patients is fourfold higher (32%) than that in patients without cirrhosis (8%) [59]. Moreover, comorbidity of COVID-19 and diabetes is also another condition that pooled data from manifold meta-analyses have proposed as a risk factor for extrapulmonary complications of COVID-19. It has been observed that diabetic mice express higher levels of ACE-2 in their renal cortex, liver, and pancreas, a finding that might be generalizable to humans and suggest a higher possibility of developing SIH in diabetic patients with COVID-19. In addition, with respect to findings that report three-year lasting hyperglycemia after recovery from *SARS-CoV-1* infection and the belief that pancreatic islets are also able to express ACE-2 receptors, it is worth considering that *SARS-CoV-2* might deal a transient or permanent damage to beta cells as well. Moreover, diabetes has been reported as a causative agent for (a) lymphocytopenia, (b) overexpression of FURIN, and (c) elevation of IL-6 production, which are associated with increased susceptibility to infection, more effective internalization of *SARS-CoV-2*, and higher chances of cytokine storm, respectively [60].

### Environmental factors

Environmental factors, such as air pollution, regional temperature, relative humidity, solar radiation, geographical features, economy, culture, and general hygiene, can affect the pattern of COVID-19 pathophysiology through four interconnected ways: (a) causing a secondary health condition or triggering a pre-existing one to relapse, (b) weakening the immune system, (c) allowing the virus to survive and spread, and (d) promoting behaviors that increase the chances of infection [61].

Unhealthy dietary habits such as the consumption of excessive carbohydrates and saturated fatty acids result in the accumulation of intrahepatic fat, which signals the incidence of NAFLD and NASH. The clinical implications of NAFLD range from an asymptomatic condition with a trace of fat accretion (> 5%) to more advanced challenges, such as a health-threatening state of steatosis that may end in NASH, liver fibrosis, cirrhosis, hepatitis, and hepatocellular cancer. The combination of *SARS-CoV-2* infection and NAFLD foretells a poor prognosis that may give rise to SIH in COVID-19 patients [62, 63].

It is also crucial to bear in mind that adequate intake of micronutrients and minerals such as iron, selenium, zinc, copper, as well as vitamins A, B family, and C should be among the principal priorities of people’s regimen. Foodstuff containing these materials interacts with different nuts and bolts of metabolism in the body and plays a pivotal role in the process of energy production. They also actively participate in counteracting oxidative reactions within organs, leading to the inhibition of inflammation and cellular stress. In addition, the intake of dietary fiber is highly recommended for the production of short-chain fatty acids, which modulate cytokine secretion and regulate the migration of immune cells. A nutrient-rich diet accompanied by essential micronutrients, minerals, and vitamins is essential for the body’s repair, maintenance of immune system robustness, and overall health, potentially resulting in a milder phenotype of respiratory distress and SIH in COVID-19 patients [45].

Lifestyle affects the quality of general health to a massive extent. A history of consistent healthy behaviors boosts innate and adaptive immunity. Low levels of physical activity have been confirmed to be a solid cause of high blood cholesterol, cardiovascular diseases, obesity, and immune-related issues. Moreover, smoking, alcohol addiction, drug abuse, overeating, and impaired sleep have also been reported to increase the rate of morbidity and mortality associated with CLDs in COVID-19, mostly by exaggerating the release of inflammatory cytokines and acute-phase proteins [7, 64–68].

**Fig. 5:**
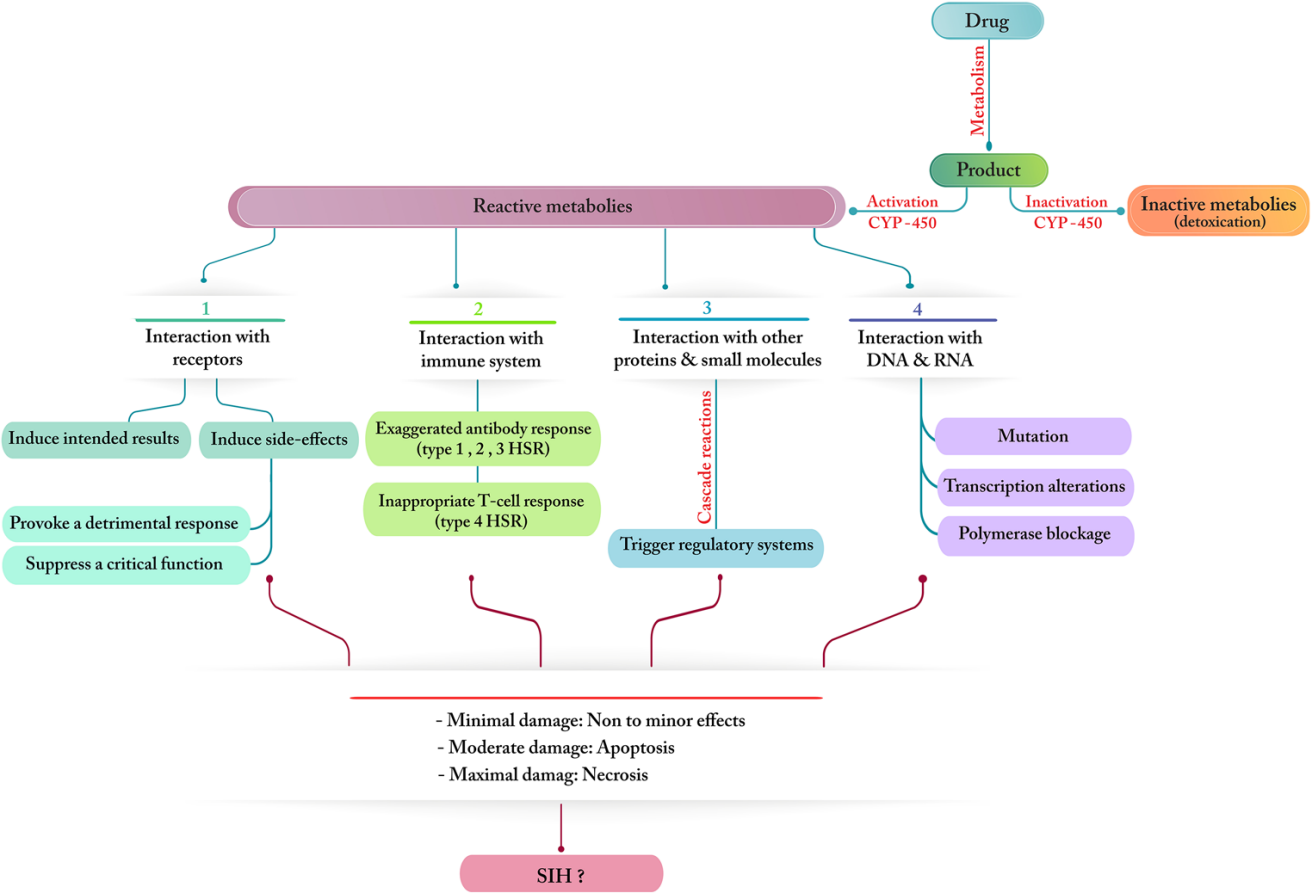
A general flowchart visualizing possible mechanisms and outcomes related to drug hepatotoxicity and their association with the progression of SIH in patients with COVID-19; (*1*) Drugs may interact with particular receptors and cause a detrimental response or suppress/shut down a crucial function. (*2*) Drugs can cause different types of hypersensitivity reactions (HSR). (*3*) Drugs may trigger certain cascade reactions that affect regulatory systems. (*4*) Drugs might also interfere with transcription and replication processes

It also appears that drugs have their own share of influence on the development of SIH, and regular monitoring along with precautionary measures should be taken to ensure that the functionality and general status of the liver in patients with COVID-19 remain optimum. Drug-induced hepatopathy is the third leading cause of liver damage after viral hepatitis and ALD/NAFLD/NASH [40]. Reports indicate that it can result in SIH after two weeks of hospitalization and drug intake. Some antiviral drugs make COVID-19 patients prone to MOF and liver injury, which addresses the potential risk of drug hepatotoxicity during the course of medication [69] (Fig. 5). Chloroquine, lopinavir/ritonavir, ribavirin, favipiravir, remdesivir, and tocilizumab are broadly prescribed candidates whose majority of metabolism is performed in the liver [70]. The literature warn that chloroquine and hydroxychloroquine can result in acute liver failure, and their use in patients with pre-existing liver diseases should be cautiously administered [57]. Furthermore, in a recent meta-analysis, the pooled incidence of drug-induced liver injury was reported to be 25.4% among COVID-19 patients, and those for whom lopinavir/ritonavir was administered, this rate (37.2%) was almost two and a half times higher than that of patients treated with remdesivir (15.2%) [71]. On the other hand, there is evidence that remdesivir, as well as systemic corticosteroids or antifungal agents, can cause elevation of aminotransferases. Ribavirin, an active agent against a wide range of RNA viruses, has also been reported to correlate with hemolysis and hepatotoxicity following cessation of its use. Moreover, COVID-19 patients who take acetaminophen to alleviate their fever are at risk of hepatic damage or even liver failure if they overdosed (Table 2) [40, 57].

**Table 2:** Environmental risk factors demonstrated in classes and types with possible mechanisms and outcomes that may contribute to SIH in patients with COVID-19

Risk factors			Possible mechanisms & outcomes	Reference
Category	Class	Type		
Environmental factors	Dietary habits	Carbohydrates	- Obesity - Insulin resistance - NAFLD & NASH - Type 2 diabetes - Liver cirrhosis	62, 63
		Saturated fatty acids		
	Unhealthy behavior	Low physical activity	- Higher chances of inflammation status - Cardiovascular diseases - Obesity - Immune-related issues - Prone to developing CLDs	64, 65
		Smoking	- Overexpression of ACE-2 by upregulation of nAChRs ^a^	66
		Alcohol drinking	- ALD - Liver cirrhosis	7
		Drug abuse	- Disturbing the activity of HPA ^b^ axis - Excessive production of ACTH ^c^ & glucocorticoids which suppress the immune system and increase the likelihood of viral invasion	67
		Impaired sleep	- Increasing the BBB ^d^ permeability - Release of inflammatory cytokines - Release of acute-phase proteins	68
		Overeating	- Increase of adipose tissue - Oversecretion of inflammatory adipokines including IL-6	63–65
	Hepatotoxic drugs	Chloroquine & hydroxychloroquine	- On-target toxicity: The pharmaceutical target site and that of the undesired effect are both the same. - Off-target toxicity: The pharmaceutical target site and that of the undesired effect are different places. - Hypersensitivity: It is the result of an unbalanced immune response to an unknown substance. - Bio-activation toxicity/antagonist interference: Molecular alterations of prodrugs may interact with receptors or regulators and interfere with their usual activity.	40, 57, 70–72
		Lopinavir/ritonavir		
		Ribavirin		
		Favipiravir		
		Remdesivir		
		Tocilizumab		

^a^Nicotinic acetylcholine receptor.

^b^Hypothalamic-pituitary-adrenal.

^c^Adrenocorticotropic hormone.

^d^Blood-brain barrier.

*Authors’ perspective: It appears that people in hot or sultry weather tend to wear face masks for a shorter period of time since the warmth is already irritating, and a physical obstacle such as face masks makes breathing even harder. On the other hand, people in cold weather are more likely to gather in groups so that the heat generated by their bodies keeps them warm. In addition, most people are likely to keep doors and windows closed to protect themselves against the low temperature outside, which results in the reduction of air velocity, accumulation of viral particles in the room, and increasing the likelihood of spreading infection. Air pollution and exposure to hazardous chemicals due to habitation in metropolitan areas or engagement in particular occupations are other reasons that might contribute to the onset of various health complications, such as cancer, or re-emergence of pre-existing diseases. These conditions can directly or indirectly affect the susceptibility of individuals to COVID-19, accelerate the progression of liver pathogenesis, and define new epidemiological patterns of the pandemic on larger scales.*

### Viral factors

The influence of viral factors, such as different variants, viral load, coinfection, virulence, and the tropism of *SARS-CoV-2* are all of great importance and relevance to our research agenda. Since the onset of the *SARS-CoV-2* pandemic, research and healthcare organizations have been dealing with an avalanche of mutations that have affected numerous biological aspects of the virus. The mutation frequency appears to be directly related to the level of virus circulation and spread, allowing it to evolve and develop competitive traits, such as enhanced virulence potency, new tropism tendencies, higher contagiousness, and more robust capabilities of evasion from neutralizing antibodies. These characteristics may set the milestones for the formation of vast waves of new outbreaks that can rapidly overtake the whole world [1, 73].

Reverse Transcription Quantitative Polymerase Chain Reaction (RT-qPCR) results revealed that patients with lower quantification cycle (Cq) values within the first 20 days of hospitalization experienced two temporary periods of elevated ALT and AST levels, indicating that a higher viral load in the early phase of the illness correlates with a higher risk of experiencing SIH. Moreover, *SARS-CoV-2* RNAemia has been observed to be linked to a greater chance of MOF development [74–77].

It also has been reported that hospitalized patients with COVID-19 show different levels of disease severity in the presence of other viral or bacterial pathogens. Coinfection with two viruses is a common event with a spectrum of diverse possibilities. *SARS-CoV-2* coinfection with influenza, *HBV*, human hepatitis C virus (*HCV*), and human immunodeficiency virus (*HIV*) have been observed in clinical cases, suggesting that they might contribute to SIH or accentuate a challenge for misdiagnosis. It has been documented that a mixed infection of *SARS-CoV-2* and *adenoviruses* may increase the risk of hypoxia, ARDS, and lymphopenia. *SARS-CoV‐2/HIV* coinfection has also been reported to be more invasive owing to immune deficiency. In addition, it has been demonstrated that *SARS-CoV-2* can cause lymphopenia and trigger the reactivation of the Epstein-Barr virus (*EBV*) and *HBV*, thus remarkably increasing the morbidity and mortality rates associated with MOF and liver involvement [16, 78]. Moreover, interactions between *SARS-CoV-2* and *Mycobacterium tuberculosis* signify the possibility of malignant hypoxic liver injury and other challenging outcomes with poor prognosis, particularly in the elderly [79].

## Conclusion

This review was gathered and composed based on the findings and data from an entire avalanche of recent meta-analyses and systemic studies to factor in a collection of various elements with possibly an influential impact on the compromised livers of COVID-19 patients or even causing the induction of liver injury associated with *SARS-CoV-2* infection. According to the collected results, the role and importance of interactions between the host, virus, and environment in this field are undeniable, and SIH is assumed to be the consequence of an extensive array of factors with a direct or indirect net effect. Overall, despite the unanimity of the outcomes in confirming that SIH is a multifactorial complication, the present area of investigation may still lack sufficient components to justify the reason for such a wide spectrum of sequelae and severity levels in COVID-19. Therefore, further studies, particularly in the context of missing factors, are required to fully understand the nuts and bolts of the mechanisms involved in the pursuit of more effective pharmaceutical approaches and the discovery of novel prognostic markers for high-risk groups.

## Data Availability

Not applicable.

## Abbreviations

ACE: Angiotensin-converting enzyme
ACTH: Adrenocorticotropic hormone
ALP: Alkaline phosphatase
ALT: Alanine aminotransferase
ANG: Angiotensin
ARDS: Acute respiratory distress syndrome
ALD: Alcoholic liver disease
AST: Aspartate aminotransferase
ATP: Adenosine triphosphate
BBB: Blood-brain barrier
BSG: Basigin
CLDs: Chronic liver diseases
COPD: Chronic obstructive pulmonary disease
COVID-19: Coronavirus disease 2019
CPE: Cytopathic effect
Cq: Quantification cycle
CRP: C-reactive protein
CRS: Cytokine release syndrome
CTSB: Cathepsin B
CTSL: Cathepsin L
CYP-450: Cytochrome P-450
DNA: Deoxyribonucleic acid
EBV: Epstein-Barr virus
GGT: Gamma-glutamyl transferase
HBV: Human hepatitis B
HCC: Hepatocellular carcinoma
HCV: Human hepatitis C
HPA: Hypothalamic-pituitary-adrenal
ICU: Intensive care unit
IL: Interleukin
LDH: Lactate dehydrogenase
MAFLD: Metabolic-associated fatty liver disease
MOF: Multiorgan Failure
nAChRs: Nicotinic acetylcholine receptor
NAFLD: Non-alcoholic fatty liver disease
NASH: Non-alcoholic steatohepatitis
NETs: Neutrophil extracellular traps
NF-κB: Nuclear factor-kappa B
NRP-1: Neuropilin 1
ORF: Open-reading frame
RAAS or RAS: Renin-angiotensin-aldosterone system
RBD: Receptor binding domain
RNA: Ribonucleic acid
ROS: Reactive oxygen species
RT-PCR: Reverse transcription polymerase chain reaction
SARS-CoV-2: Severe acute respiratory syndrome coronavirus 2
SIH: SARS-CoV-2-induced hepatopathy
TBIL: Total bilirubin
TMPRSS-2: Transmembrane protease serine 2
TNF-α: Tumor necrosis factor-alpha
